# Global quantification of newly synthesized proteins reveals cell type- and inhibitor-specific effects on protein synthesis inhibition

**DOI:** 10.1093/pnasnexus/pgad168

**Published:** 2023-05-19

**Authors:** Kejun Yin, Ming Tong, Suttipong Suttapitugsakul, Senhan Xu, Ronghu Wu

**Affiliations:** School of Chemistry and Biochemistry and the Petit Institute for Bioengineering and Bioscience, Georgia Institute of Technology, Atlanta, GA 30332, USA; School of Chemistry and Biochemistry and the Petit Institute for Bioengineering and Bioscience, Georgia Institute of Technology, Atlanta, GA 30332, USA; Novo Nordisk R&D Centre, Beijing 102206, China; School of Chemistry and Biochemistry and the Petit Institute for Bioengineering and Bioscience, Georgia Institute of Technology, Atlanta, GA 30332, USA; Department of Surgery, Beth Israel Deaconess Medical Center, Harvard Medical School, Boston, MA 02215, USA; School of Chemistry and Biochemistry and the Petit Institute for Bioengineering and Bioscience, Georgia Institute of Technology, Atlanta, GA 30332, USA; School of Chemistry and Biochemistry and the Petit Institute for Bioengineering and Bioscience, Georgia Institute of Technology, Atlanta, GA 30332, USA

**Keywords:** bioorthogonal chemistry, protein synthesis inhibition, protein inhibition efficiency, mass spectrometry, multiplexed proteomics, newly synthesized proteins

## Abstract

Manipulation of protein synthesis is commonly applied to uncover protein functions and cellular activities. Multiple inhibitors with distinct mechanisms have been widely investigated and employed in bio-related research, but it is extraordinarily challenging to measure and evaluate the synthesis inhibition efficiencies of individual proteins by different inhibitors at the proteome level. Newly synthesized proteins are the immediate and direct products of protein synthesis, and thus their comprehensive quantification provides a unique opportunity to study protein inhibition. Here, we systematically investigate protein inhibition and evaluate different popular inhibitors, i.e. cycloheximide, puromycin, and anisomycin, through global quantification of newly synthesized proteins in several types of human cells (A549, MCF-7, Jurkat, and THP-1 cells). The inhibition efficiencies of protein synthesis are comprehensively measured by integrating azidohomoalanine-based protein labeling, selective enrichment, a boosting approach, and multiplexed proteomics. The same inhibitor results in dramatic variation of the synthesis inhibition efficiencies for different proteins in the same cells, and each inhibitor exhibits unique preferences. Besides cell type- and inhibitor-specific effects, some universal rules are unraveled. For instance, nucleolar and ribosomal proteins have relatively higher inhibition efficiencies in every type of cells treated with each inhibitor. Moreover, proteins intrinsically resistant or sensitive to the inhibition are identified and found to have distinct functions. Systematic investigation of protein synthesis inhibition in several types of human cells by different inhibitors provides valuable information about the inhibition of protein synthesis, advancing our understanding of inhibiting protein synthesis.

**Significance Statement**

Protein synthesis inhibition is extensively utilized in bio-related research, and different inhibitors have been investigated and applied to various studies. However, the synthesis inhibition efficiencies of different proteins even by one inhibitor and the differences among different inhibitors remain to be explored. We systematically study protein synthesis inhibition and several popular inhibitors through comprehensive quantification of newly synthesized proteins. Besides the overall protein inhibition efficiencies by these inhibitors, the efficiencies of individual proteins by each inhibitor are obtained. The same inhibitor causes dramatic differences in synthesis inhibition of different proteins in the same cells. Furthermore, protein synthesis inhibition shows cell type- and inhibitor-specific effects. This work provides valuable information to the scientific community, helping scientists to choose a right inhibitor.

## Introduction

Protein synthesis is essential to nearly every biological process, including cell growth and proliferation. Dysregulation of protein synthesis is related to various diseases, such as cancer and cognitive disorders (1). Hence, understanding and manipulating protein synthesis are critical to expand our knowledge of protein functions, cellular activities, and disease mechanisms. Multiple protein synthesis inhibitors with distinct mechanisms have been extensively studied and utilized in biological and biomedical research fields (2). Notably, cycloheximide (CHX), blocks the E-site in the 60S ribosome (3). Puromycin (Puro) mimics the aminoacyl-tRNAs and is incorporated into nascent polypeptide chains, causing the early release of peptides by blocking the elongation of the nascent polypeptides (4). Anisomycin (ANM) is a competitive inhibitor of peptide bond formation and stops peptide elongation (5). They have been widely employed in bio-related research and demonstrated great potential for treating cancer and other diseases (6, 7).

These inhibitors are commonly used to globally inhibit protein synthesis, and they can result in different inhibition efficiencies for the synthesis of individual proteins. For example, it was reported that ANM had the inhibition efficiency of only 65% for CYP2B1 when >95% of total protein synthesis was terminated in rat hepatocytes, but Puro inhibited ∼95% CYP2B1 expression while achieving a relatively low overall protein synthesis inhibition (8). Systematic investigation of the efficiencies of protein synthesis inhibition can facilitate our understanding of the regulation of the protein translation process and these inhibitors. However, despite the success of measuring the inhibition efficiencies of single proteins, systematic evaluation of protein synthesis inhibitors is exceptionally challenging. Even though the binding affinity of each inhibitor with its target and/or the ribosome can be measured, it is not directly reflective of its inhibition efficiency for protein synthesis and cannot reveal the inhibition efficiencies for individual proteins.

Recently, the development of ribosome profiling (Ribo-Seq) allows for the transcriptome-scale translation efficiency evaluation through sequencing and quantifying the mRNA fragments protected by the ribosomes during the translation process. This method was utilized to investigate the effects of inhibitors on the protein translation process (9). However, this method is not for direct measurement of the production of protein synthesis. Furthermore, stalled ribosomes are not excluded in the Ribo-Seq workflow, making this method less effective in studying protein synthesis inhibition (10). It is also impractical to measure the synthesis inhibition efficiencies using antibody-based approaches because newly synthesized proteins normally contain identical epitopes with their preexisting counterparts. Moreover, antibody-based approaches are not ideal for large-scale analysis. Newly synthesized proteins are the direct products of protein synthesis by the ribosome, and thus, comprehensive and quantitative analysis of these proteins is perfectly suited to systematically study and evaluate protein synthesis inhibition and different inhibitors. Currently, mass spectrometry (MS)-based proteomics enables us to globally analyze proteins and their modifications (11–17). However, comprehensive analysis of newly synthesized proteins is not trivial because these proteins are typically present in low abundance and masked by many highly abundant preexisting proteins during MS analysis. Furthermore, they need to be distinguished from their preexisting copies.

In this work, we systematically and quantitatively study the synthesis inhibition efficiencies of proteins by different popular inhibitors (CHX, Puro, and ANM) through global analysis of newly synthesized proteins in four types of widely used human cells (A549, Jurkat, MCF7, and THP-1 cells). Comprehensive and quantitative analysis of these proteins was achieved by integrating metabolic labeling, bioorthogonal chemistry-based enrichment, and multiplexed proteomics. Moreover, we incorporated a boosting approach to enhance the identification of newly synthesized proteins with low abundance. In total, >5,000 newly synthesized proteins were accurately quantified, and their inhibition efficiencies were obtained. The results demonstrate that protein synthesis inhibition efficiencies are markedly different among the cell types even for the same inhibitor, and the efficiencies for individual proteins in the same type of cells by the same inhibitor also vary dramatically. Among these inhibitors, Puro is the most nondiscriminatory, while CHX is relatively less effective in inhibiting the synthesis of ribosomal proteins despite its overall high potency and popularity. The correlations between the inhibition efficiencies of individual proteins and their various physicochemical and biological properties are further investigated. This work provides unprecedented and valuable information about the inhibition of protein synthesis, facilitating a better understanding of protein synthesis regulation. Both the inhibition efficiencies of individual proteins by each inhibitor and the different inhibition efficiencies of the same proteins in different cell types are beneficial for future biomedical studies.

## Results

### Quantification of newly synthesized proteins and measurement of the protein synthesis inhibition efficiencies

Quantification of newly synthesized proteins in cells with the treatment of each inhibitor can provide straightforward evidence for protein synthesis inhibition. Previously, methods such as bioorthogonal noncanonical amino acid tagging (BONCAT) and multiplex isobaric tagging/noncanonical amino acid tagging (MITNCAT) were developed to investigate newly synthesized proteins (18–23). Most of them utilize methionine analogs, i.e. azidohomoalanine (AHA) or homopropargylglycine (HPG), to pulse-label cells so that newly synthesized proteins are modified with a clickable handle, allowing for their enrichment. However, due to the low abundance of newly synthesized proteins, especially when a protein synthesis inhibitor is used, a relatively long labeling time is usually required to accumulate a sufficient amount of labeled newly synthesized proteins. When evaluating protein synthesis inhibitors, a longer labeling time is not ideal because protein expressions could be altered when the cells respond to the inhibition. Thus, we chose a relatively short inhibitor treatment time (1.5 h) and a pulse-labeling time of 1.0 h, which can minimize undesired cellular responses, while it is long enough to inhibit protein synthesis as reported previously (9, 24, 25).

**Fig. 1.**
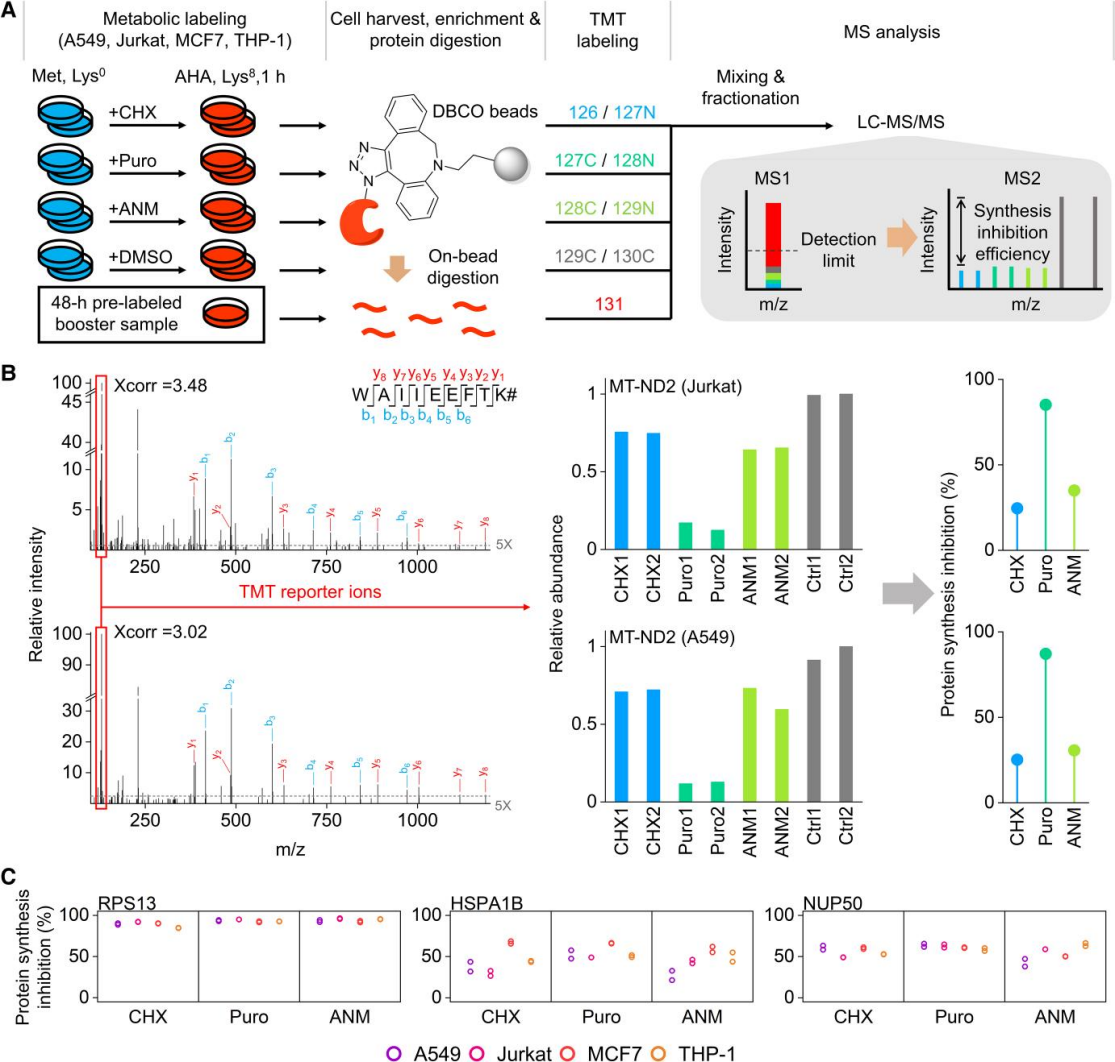
Experimental design and example peptide and protein quantification. A) Experimental procedure for global analysis of newly synthesized proteins with the treatment of CHX, Puro, ANM, or DMSO (as the control), respectively. B) An example of the tandem mass spectra of WAIIEEFTK# (# refers to Lys^8^) together with the relative intensities of the reporter ions in Jurkat and A549 cells. The dashed line represents the intensity at five times noise level. The calculated protein synthesis inhibition efficiencies of MT-ND2 are also provided. C) Examples of the protein synthesis inhibition efficiencies with different inhibitors in different types of cells.

To enhance the identification of newly synthesized proteins with a short treatment time, a boosting approach was integrated into the current workflow. Based on a previous report, if the amount of the booster sample is too high (e.g. >20×), the quantification accuracy may be affected (26). Given that the portion of protein synthesized within 1.0 h in cells is very low, the fully stable isotope labeling by/with Amino acids in cell culture (SILAC)-labeled proteome may not be the best to serve as the boosting sample. Hence, we employed a 48-h prelabeled sample as the booster sample (Fig. 1A), which gives about five times signal enhancement for the majority of detected newly synthesized peptides (Dataset S1). The signals of the low-stoichiometry, SILAC-labeled peptides from newly synthesized proteins are greatly enhanced at both the MS1 and MS2 levels while achieving reasonably high reproducibility (low coefficients of variance [CVs]) on protein intensities between the replicates. The boosting approach can greatly improve the identification of newly synthesized proteins because many low-abundance ones may be below the detection limit of MS.

To systematically evaluate the commonly used protein synthesis inhibitors (CHX, ANM, and Puro), we globally identified and quantified newly synthesized proteins in various types of human cells (A549, Jurkat, MCF7, and THP-1) with each inhibitor (Fig. 1A). The AHA labeling of newly synthesized proteins allows for their following enrichment through the click reaction (27, 28), and the heavy-lysine labeling enables us to unambiguously distinguish them from preexisting ones. Digested peptides from enriched newly synthesized proteins were reacted with the tandem mass tag (TMT) reagents, and peptides in each group were combined, fractionated, and analyzed by liquid chromatography (LC)–MS.

To better evaluate the protein synthesis inhibition and different inhibitors, gel experiments were performed to choose the concentration of each inhibitor for the proteomics experiments (Figs. S1A and B). The concentrations were carefully chosen to have the same overall inhibition efficiencies of total proteins (∼95%) for these inhibitors, allowing for fair comparison of the inhibitors and the inhibition efficiencies of individual proteins by different inhibitors. Later on, in the proteomics experiments, the efficiencies were found to be ∼80% in all cases (Fig. S1C). The difference may be because the gel experiment is semiquantitative. Furthermore, fragments of newly synthesized proteins with smaller sizes may not remain in the size range detected in the gel experiment. However, this does not affect our analysis because the overall inhibition efficiencies among these inhibitors are still comparable. The inhibition efficiency of each protein was obtained as described in the Materials and methods section (Datasets S1 and S2).

As an example, CHX and ANM achieved ∼30% of the synthesis inhibition of NADH–ubiquinone oxidoreductase chain 2 (MT-ND2) in two types of cells (Jurkat and A549 cells), while Puro reached >85% inhibition of this protein in the same types of cells (Fig. 1B). This observation agrees with the fact that protein synthesis in the mitochondrion is more resistant to the inhibition by CHX and ANM (cytosolic ribosome inhibitors (29, 30)), and thus, a low efficiency is expected for MT-ND2, one of 13 proteins synthesized in the mitochondrion (31). A similar trend was observed for other proteins synthesized in the mitochondrion. For instance, the synthesis of NADH–ubiquinone oxidoreductase chain 1 (MT-ND1) was barely affected by CHX and ANM in A549 and Jurkat cells, while Puro reached nearly 50% inhibition efficiency. Puro also showed higher efficiencies in inhibiting the synthesis of mitochondrially encoded cytochrome b (MT-CYB) in Jurkat, MCF7, and THP-1 cells. The inhibition efficiencies differ considerably among quantified proteins under the treatments of different inhibitors in different types of cells, which clearly demonstrated that each inhibitor unevenly interrupted the syntheses of individual proteins, as indicated by the examples in Fig. 1C. Additionally, the CVs of protein intensities were reasonably low between the replicates (Fig. S1D).

### An overview of the protein synthesis inhibition efficiencies by each inhibitor

**Fig. 2.**
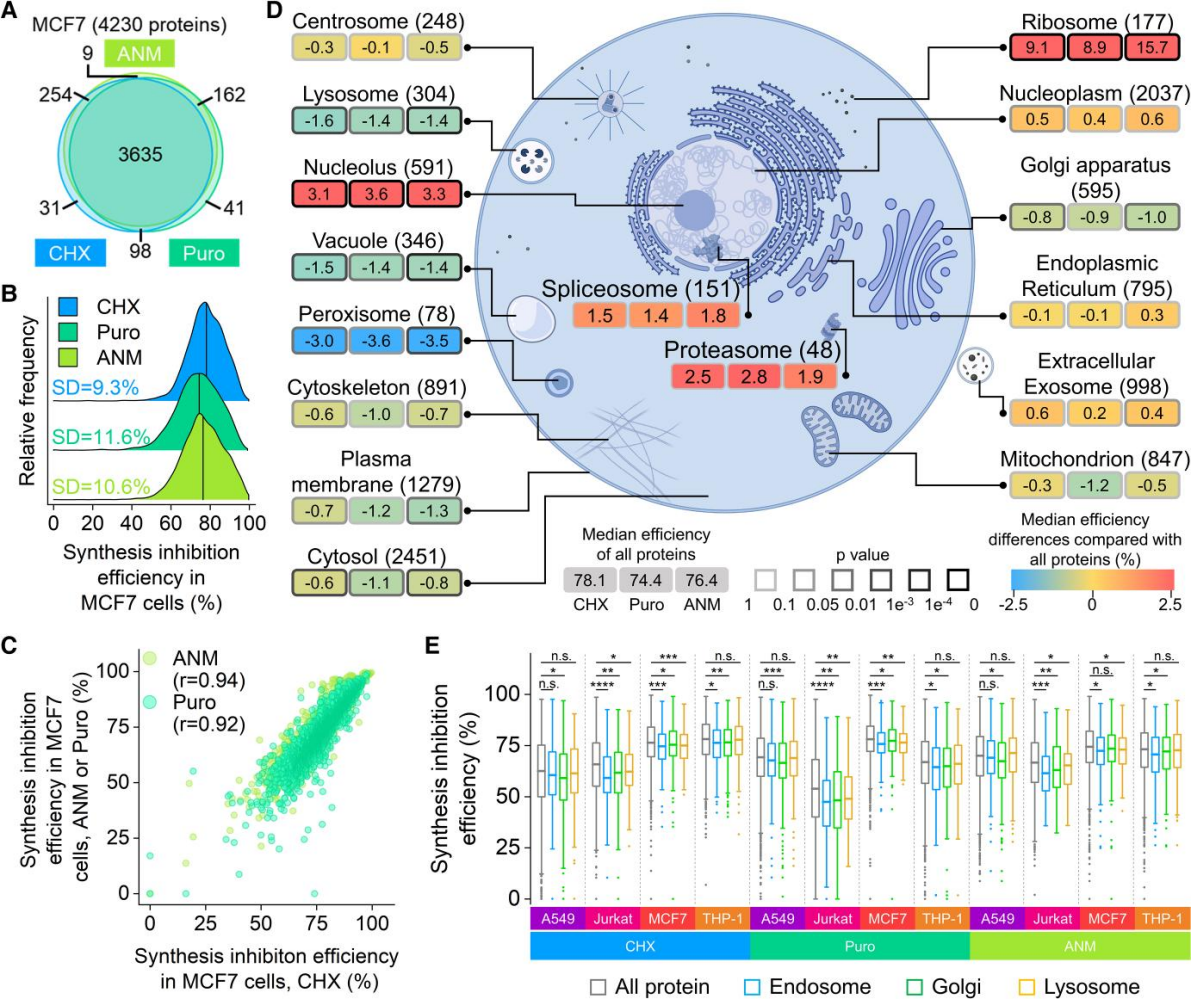
Protein synthesis inhibition efficiencies in MCF7 cells. A) Overlap of the proteins quantified in three inhibitor treatments in MCF7 cells. B) Distribution of the protein synthesis inhibition efficiencies in MCF7 cells with each inhibitor. The median value of each group of data set is shown as the black drop line. The SD of each data group is also presented. C) Correlations between the protein synthesis inhibition efficiencies of the three inhibitors in MCF7 cells. D) The inhibition efficiencies of proteins in different cellular components and protein complexes among three inhibitors in MCF7 cells. The number of proteins quantified in each category is presented in parentheses. The color of the box represents the median inhibition efficiency difference compared with the median efficiency of all quantified proteins by each inhibitor, and the value difference is also shown in each box. The darkness of the box border indicates the significance of the difference (determined by the K–S test). Proteins with more than one cellular component annotations were kept in each component. E) Synthesis inhibition efficiency distributions of quantified proteins in the Golgi, lysosome, and endosome and comparison with all quantified proteins (centerline, median; box limits, the first and third quartiles; whiskers, 1.5 interquartile range; n.s.-no significant difference, *P < 0.05, **P < 0.01, ***P < 0.001, and ****P < 0.0001). Statistical significance was determined by the K–S test.

Although the mechanisms of these three inhibitors have been well studied, their inhibition efficiencies for individual proteins have never been systematically quantified and evaluated. Based on the total intensities of the reporter ions for each treatment, the overall inhibition efficiencies in MCF7 cells are 85.9%, 85.9%, and 86.7% for CHX, Puro, and ANM, respectively (Fig. S1C). Overall, the inhibition efficiencies of 4,230 proteins in MCF7 cells were quantified (Fig. 2A). With the treatment of each inhibitor, the inhibition efficiencies span a wide range across quantified proteins, which demonstrates that the synthesis of individual proteins is not evenly inhibited by the same inhibitor (Fig. 2B). Although this might be expected, unveiling this bias systematically and quantitatively at the proteome level is of great importance, despite the previous mRNA-based translatome analysis (32). The SD of each data group is ∼10%, with CHX having the least variation. The efficiencies of individual proteins by three inhibitors are highly correlated in MCF7 cells (Fig. 2C).

To systematically evaluate the inhibition efficiencies of individual proteins in different cellular components and protein complexes by each inhibitor, we performed Gene Ontology (GO) analysis using the Database for Annotation, Visualization, and Integrated Discovery (DAVID) (33) and clustered proteins quantified in MCF7 cells based on their subcellular locations. Figure 2D shows the number of quantified proteins in each cellular component or protein complex, with the differences between their median inhibition efficiencies and the median value of all quantified efficiencies by each inhibitor (details in Dataset S3). The significance of each comparison determined by the Kolmogorov–Smirnov (K–S) test was indicated as the border darkness of each box. The inhibition efficiencies of individual proteins vary dramatically across different cellular components or protein complexes.

Generally, the performances of the three inhibitors are similar for proteins in the same cellular components and protein complexes (Fig. 2D). Considerable variations of the inhibition efficiencies are found among proteins in different subcellular locations. Typically, all three inhibitors efficiently inhibited the synthesis of nucleolar and ribosomal proteins. The nucleolus is the primary location for the synthesis of ribosomal proteins and the assembly of the ribosomes (34). The current results suggested that besides directly causing functional loss of the ribosomes, these inhibitors suppress the ribosome biogenesis. However, proteins in the peroxisome, the lysosome, and the vacuole are relatively less inhibited by all three inhibitors.

We further analyzed the inhibition efficiencies of proteins in four types of cells by CHX. For different types of cells treated with the same inhibitor, much more variations of the inhibition efficiencies were observed (Fig. S2). For example, plasma membrane and mitochondrial proteins are notably less inhibited in Jurkat cells, but not in the other three cell types. Proteins in the spliceosome are more inhibited in two types of suspension cells, while in A549 and MCF7 cells, they have no significant differences compared with all quantified proteins. The proteasome subunits were less inhibited than other proteins in the suspension cells, respectively, while they were more inhibited in the two types of adhesion cells. These differences may be attributed to the characteristics of each cell type, which are discussed below. Besides those variations, proteins in some cellular compartments displayed a similar trend across different types of cells. Similarly, nucleolar and ribosomal proteins were highly inhibited in all tested cell types. This further demonstrates that the synthesis of proteins involved in the translation processes was highly inhibited.

Proteins in the Golgi were significantly less inhibited in all four cell types by CHX and all other experiments except for the Puro treatment in MCF7 cells (Fig. 2E). The Golgi is the center of the complex endomembrane system, and we further analyzed other compartments in this system. Proteins in the endoplasmic reticulum (ER) did not show any trend (Figs. 2D and S2). Lysosomal proteins presented lower efficiencies in Jurkat and MCF7 cells by three inhibitors while insignificant in the other two types of cells. Proteins in the endosome were less inhibited, except for A549 cells. Overall, most proteins in the Golgi, the lysosome, and the endosome have relatively low inhibition efficiencies.

**Fig. 3.**
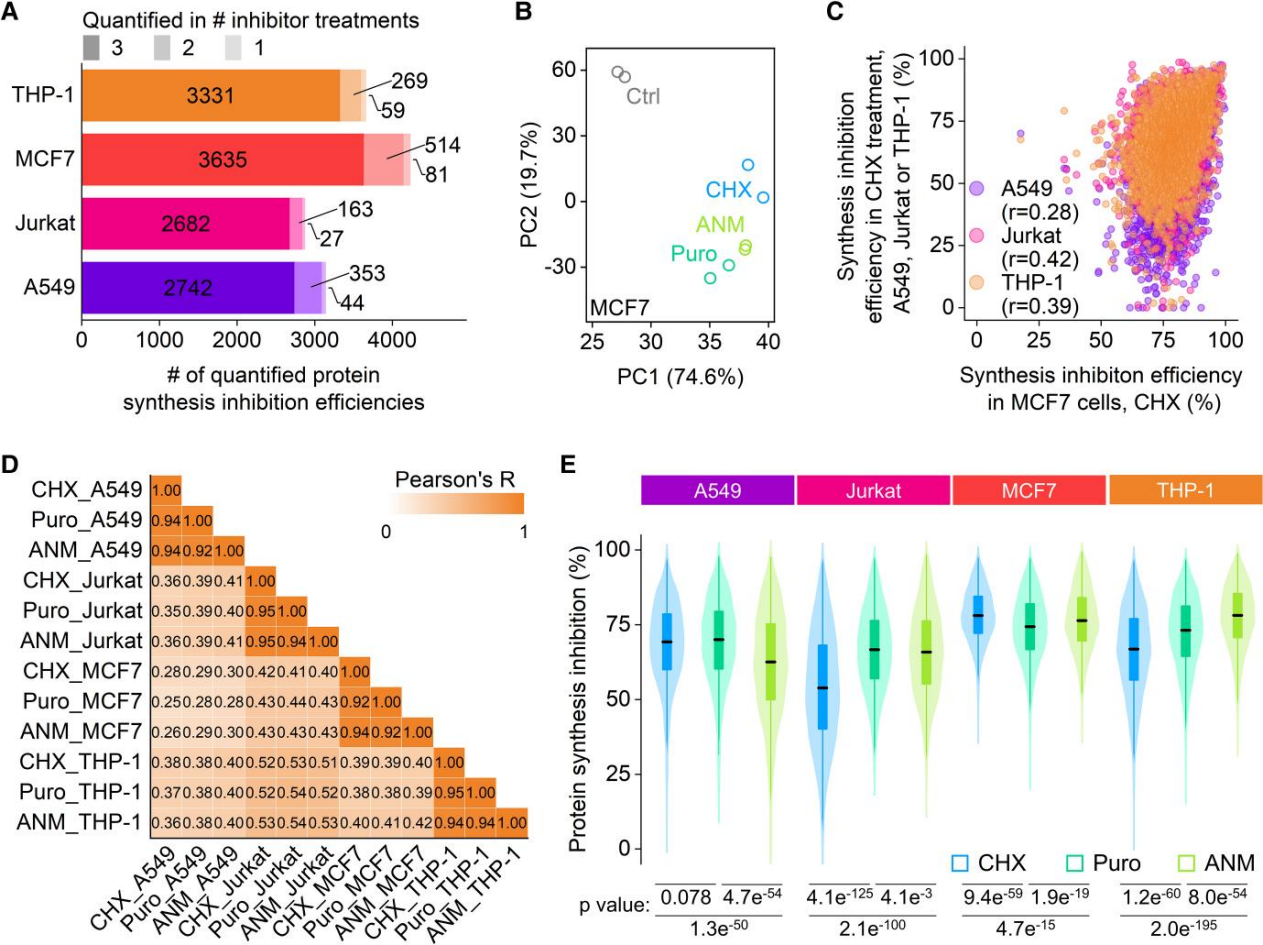
Protein synthesis inhibition efficiencies in different types of cells with different inhibitors. A) The number of quantified protein synthesis inhibition efficiencies in each cell type. B) Principal component analysis of the replicated samples under each treatment and the control samples in MCF7 cells. C) Correlations of the inhibition efficiencies of individual proteins in four cell types treated with CHX. D) Correlation matrix of all protein synthesis inhibition efficiencies measured in all experiments. Pearson's correlation coefficient (*R*) is shown for each comparison. E) Distributions of the protein synthesis inhibition efficiencies are displayed as the violin plots (centerline, median; box limits, the first and third quartiles; whiskers, 1.5 interquartile range). Statistical significance was determined by the K–S test.

Totally, 5,617 newly synthesized proteins were quantified (Figs. 3A and S3A). The samples with different treatments were grouped using principal component analysis (Figs. 3B and S3B). The results from the duplicate experiments using each inhibitor were well clustered together and clearly separated from the control ones. Unlike the comparison among different inhibitors in the same type of cells, the correlations of the inhibition efficiencies for individual proteins by the same inhibitor in different types of cells are much lower (Fig. 3C and D), indicating that the inhibition of protein synthesis is highly cell-type dependent.

The distribution of the synthesis inhibition efficiencies under each treatment showed that different inhibitors had various inhibitions for different proteins in the same type of cells, and when compared between the cell types, each inhibitor also resulted in different efficiency distributions (Fig. 3E). For instance, in A549 cells, ANM had significantly lower efficiencies than CHX and Puro, but it had the highest efficiencies in THP-1 cells. CHX demonstrated the lowest efficiencies in Jurkat and THP-1 cells, while it had the highest efficiencies in MCF7 cells. Puro exhibited the least inhibition efficiency variations across all cell types among the three inhibitors.

To better understand proteins with various inhibition efficiencies across all experiments, we defined proteins with top 25% efficiencies as sensitive to a specific inhibitor in one cell type (i.e. one experiment). Similarly, those with the bottom 25% efficiencies were annotated as resistant proteins (Dataset S4). In total, 2,472 and 2,937 proteins were annotated as sensitive or resistant at least once in all experiments, respectively. Among them, 719 proteins were annotated as both sensitive and resistant in different experiments, indicating that despite many proteins with similar efficiencies across all experiments, the efficiencies of some proteins were inhibitor and/or cell-type specific. GO analysis showed that resistant proteins were enriched in terms related to protein folding, mitochondrial matrix, and protein transportation (Fig. S3C and Dataset S5). Ribosomal proteins and rRNA processing proteins were highly enriched in the sensitive protein group, which further indicates that cells stopped the production of the translation machinery components under the inhibitor treatment.

### Properties of proteins intrinsically sensitive or resistant to the inhibition

The regulation of protein synthesis is critical for proteostasis. Dysregulation of protein synthesis is related to multiple diseases, and protein synthesis inhibitors are powerful for disease treatment (35). However, proteins intrinsically resistant or sensitive towards different inhibitors are still largely unknown. The investigation of these three inhibitors with distinct mechanisms in four types of cells allows us to systematically understand the inhibition of protein synthesis with minimal inhibitor and cell-specific effects.

**Fig. 4.**
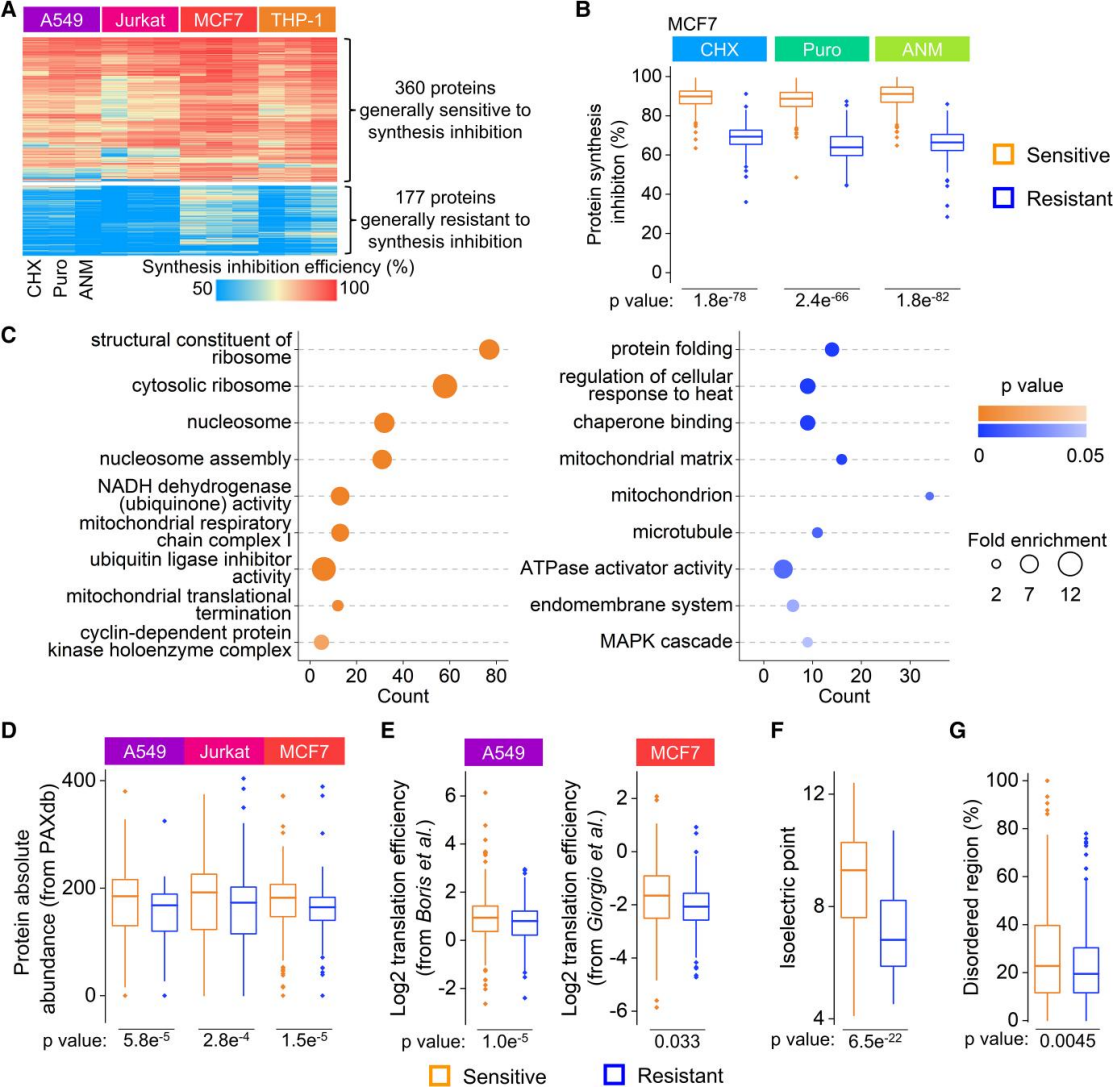
Evaluating the properties of proteins intrinsically sensitive or resistant to the inhibition. A) Heatmap of the synthesis inhibition efficiencies for proteins being annotated as sensitive or resistant. B) Distributions of the synthesis inhibition efficiencies for proteins sensitive or resistant to the inhibition in MCF7 cells. C) GO analysis of proteins that are annotated as sensitive (left) and resistant (right). Some selected enriched terms are presented. D) Distributions of the abundances of proteins sensitive or resistant to the inhibition. The protein abundances in each type of cells are from PAXdb. E) Distributions of the translation efficiencies for proteins sensitive or resistant to the inhibition. The translation efficiencies in A549 cells are from Giorgio et al., and those in MCF7 cells are from Boris et al. F, G) Comparisons of isoelectric point F) and disorderness G) between proteins sensitive and resistant to the inhibition. (For boxplots: center line, median; box limits, the first and third quartiles; whiskers, 1.5 interquartile range. Statistical significance was determined by the K–S test.)

We next performed more analysis to further understand proteins sensitive or resistant to the inhibition. First, proteins annotated as sensitive/resistant in two out of three treatments in one cell type were selected to exclude inhibitor-specific effects. Then proteins annotated as sensitive/resistant in three out of four cell types were further selected as generally sensitive/resistant to synthesis inhibition to minimize cell type-specific effects (Fig. S4A). Overall, 360 proteins were intrinsically sensitive to the inhibition, and 177 were resistant (Fig. 4A). These two groups of proteins exhibited dramatic and significant inhibition efficiency differences in all experiments (Figs. 4B and S4B). For those sensitive to the inhibition, proteins related to the ribosome and the nucleosome assembly were enriched, indicating that the synthesis of proteins related to transcription and translation processes was highly impaired under the inhibition (Fig. 4C and Dataset S6). Furthermore, proteins that participated in cellular respiration processes were overrepresented. Among resistant proteins, those related to folding stress response were significantly enriched, emphasizing the importance of chaperones during the protein synthesis inhibition. Mitochondrial proteins were overrepresented as well. A possible explanation for protein resistance to the inhibition is that these proteins are more important for cells to respond to the protein synthesis inhibition or maintain cell viability through evolution. For instance, chaperone proteins were highly resistant to all three inhibitors (Fig. 4C) and are known to be crucial for maintaining essential cellular functions. In this case, the inhibition efficiencies contain more valuable information beyond the inhibitor evaluation.

We analyzed the abundances of proteins sensitive or resistant to the inhibition (Dataset S7 and Fig. 4D, protein abundances from PAXdb (36)) and found that sensitive proteins are more abundant than resistant ones. The results indicate that proteins with higher synthesis rates can be inhibited more effectively. Furthermore, we compared the current inhibition efficiencies with the translation efficiencies from the published Ribo-Seq data sets (37, 38), which are very relevant to protein synthesis because the translation efficiency is defined as the ratio of mRNA being translated in the ribosomes compared with all mRNA copies of a gene in cells. Sensitive proteins have significantly higher translation efficiencies (Fig. 4E). We further investigated other properties of proteins sensitive and resistant to the inhibition, including isoelectric point, hydrophobicity, number of interactors, and disordered regions (Dataset S7). Proteins sensitive to the inhibition have significantly higher isoelectric points (Fig. 4F), more disordered regions (Fig. 4G), less hydrophobic based on their GRAVY scores (Fig. S4E), and have more interactors in cell (Fig. S4D) (39). mRNA serves as a template for protein synthesis, and their abundances in cells may be related to proteins sensitive or resistant to the inhibitions. We compared the baseline mRNA expression between sensitive and resistant proteins across four types of cells (Dataset S7, mRNA data from DepMap portal). The results demonstrated that proteins sensitive to the inhibition had significantly higher baseline mRNA levels (Fig. S4C), and this trend exists in every cell type studied here. The mRNA level is generally considered to be positively correlated with the protein synthesis rate.

### Differential inhibition sensitivity of proteins across cell types correlated with cell-type characteristics

**Fig. 5.**
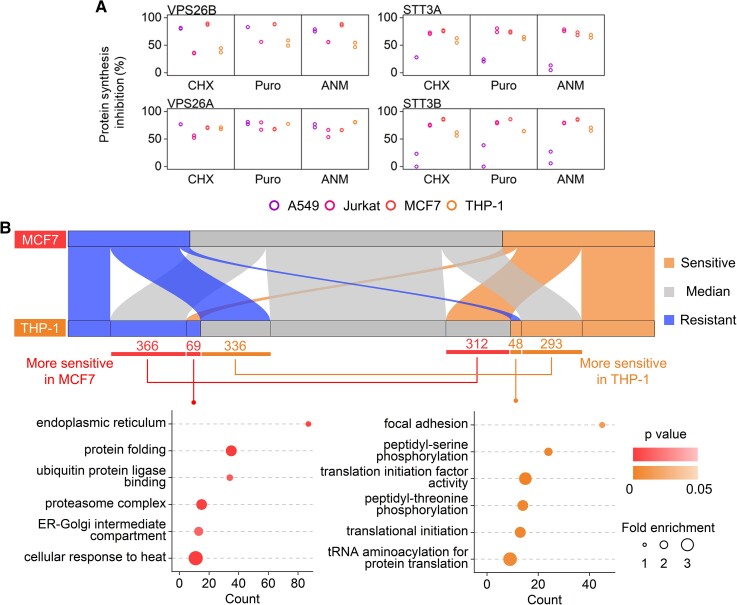
Cell type-specific differences of protein synthesis inhibitions. A) Selected example proteins with different sensitivities to the inhibitors in different types of cells. B) Sankey diagram based on the assignment of a specific sensitivity annotation (sensitive, median, and resistant) in comparison between MCF7 and THP-1 cells. Selected GO terms enriched among proteins more sensitive in MCF7 or THP-1 cells are also presented with all quantified proteins as the background.

One of the most remarkable differences among the four types of cells in this study is that A549 and MCF7 cells are adhesive, while Jurkat and THP-1 cells are suspension ones. We investigated how the characteristic of each cell type could influence protein inhibition efficiencies. As an example, vacuolar protein sorting 26 homolog B (VPS26B) exhibited great resistance to the inhibition in two types of suspension cells, but it was sensitive in A549 and MCF7 cells (Figs. 5A and S5A). Immune cells are known to be the most motile in the human body, and their adhesive interactions are highly regulated in immunological responses (40). VPS26 is a subunit of the retromer complex, which recycles transmembrane cargos from the endosome to the plasma membrane and the Golgi (41), including many proteins related to cell adhesion (42). VPS26 is associated with the retromer cargo selection, and the two paralogs VPS26A and VPS26B in mammalian cells have different specificities despite their high sequence similarity (43). However, for VPS26A, no cell type-specific inhibition was observed (Fig. 5A), suggesting that the cell type-specific inhibition of VPS26B could be related to its unique function. As another example, STT3A and STT3B, two oligosaccharyltransferase isoforms responsible for protein *N*-glycosylation (44), also demonstrate the cell-specific inhibition differences. STT3A and STT3B had extraordinarily high resistance to all inhibitors in A549 cells but not in the other types of cells (Fig. 5A). Because *N*-glycosylation is essential to mammalian cells, it is attractive to investigate further why STT3A and STT3B had the distinct inhibition sensitivities among the cell types.

We found all proteins with great differences between any two types of cells and performed GO analysis (Fig. S5B). Each pair of comparisons includes 57 to 140 proteins, and their functions may correlate with the features of the cell types. While the results are interesting, the analysis was limited by the relatively low numbers of proteins. To better characterize the inhibition efficiency differences between cell types, we expanded to all proteins with their sensitivity annotation changes between two cell types. As an example, the comparison between MCF7 and THP-1 cells was performed (Fig. 5B and Dataset S8), and 747 proteins were more sensitive in MCF7 cells. These proteins are enriched in protein quality control-related terms, like protein folding and proteasome complex. Also, proteins in the ER and the ER–Golgi intermediate compartments were more sensitive in MCF7 cells, indicating that these proteins may be less essential in MCF7 cells than in THP-1 cells. This could be because that protein secretion through the classical secretory pathway is more active and more critical in immune cells (45), such as THP-1 cells. For THP-1 cells, 677 proteins were more sensitive, among which focal adhesion proteins were highly enriched, consistent with the fact that proteins related to cell adhesion are more vital in MCF7 cells than THP-1 cells.

### Differential synthesis inhibition sensitivity of proteins across the inhibitors

**Fig. 6.**
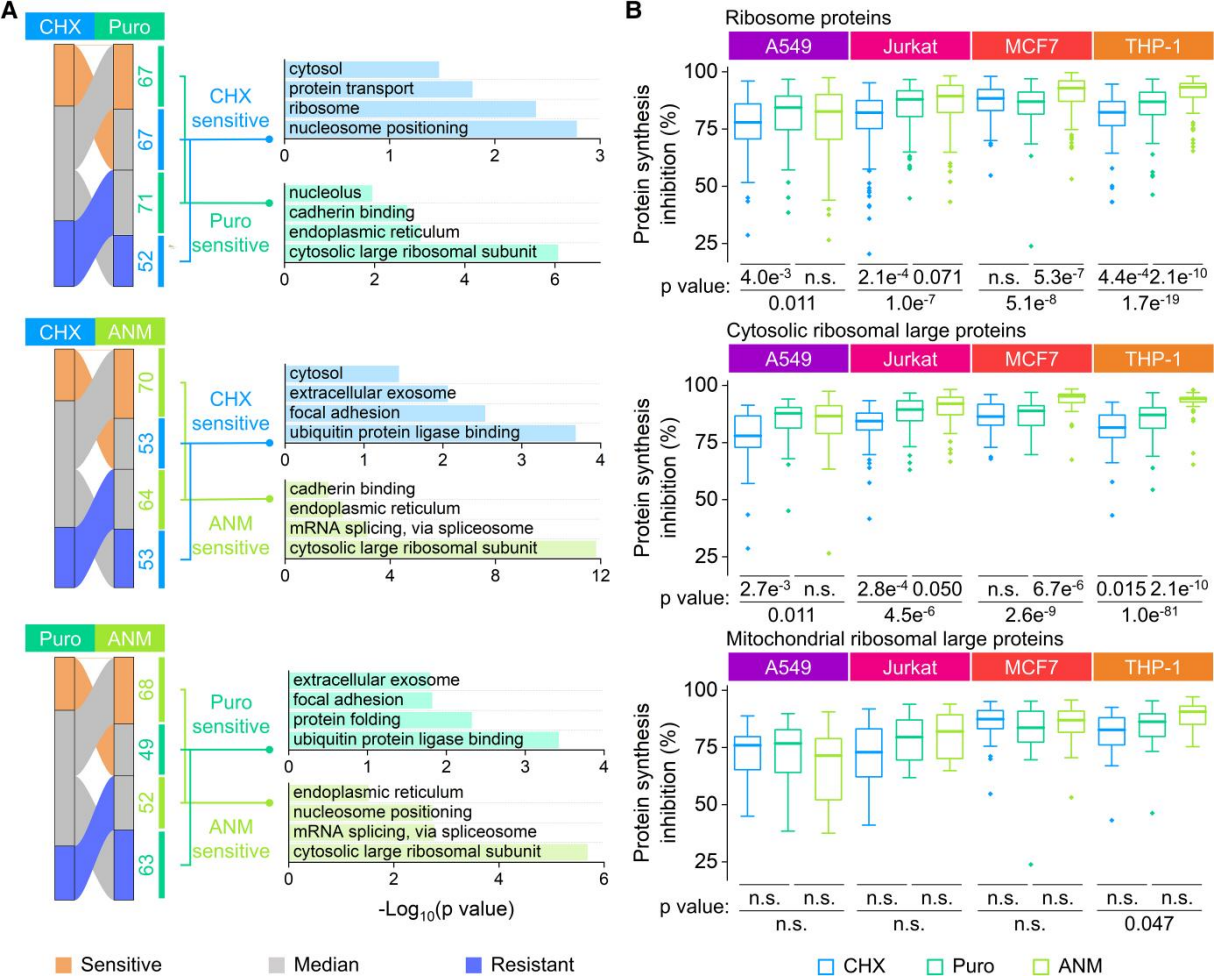
Inhibitor-specific differences in the inhibition sensitivity of proteins. A) Sankey diagrams show proteins with different sensitivity annotations between any two inhibitors. The numbers of proteins in each category are presented next to the relative nodes. Selected GO terms enriched among proteins in each group are included. B) All quantified inhibition efficiencies of ribosomal proteins (top), cytosolic large ribosomal proteins (middle), and mitochondrial large ribosomal proteins (bottom). (Centerline, median; box limits, the first and third quartiles; whiskers, 1.5 interquartile range. Statistical significance was determined by the K–S test. “n.s.” means *P* > 0.1.)

The performance variation of the three inhibitors has been documented for single proteins (8), but systematic comparison at the proteome level has yet to be reported. To exclude differences caused by various cell types, we applied the above criteria: proteins sensitive/resistant to one inhibitor in three of four cell types. More than 300 proteins were annotated as sensitive for each inhibitor, and nearly 200 were resistant (Fig. S7A). These differences may be at least partially attributed to the differences in the modes of action of the three inhibitors. For instance, ER proteins had different inhibition efficiencies between the CHX and ANM treatments in this work (Fig. 6A). Previously, Ogg et al. observed that when disrupting newly synthesized protein translocation from the cytosol to the ER, cells were stressed because of the accumulation of premature polypeptides in the cytosol. CHX relieved such stress by reducing newly synthesized protein production, while ANM failed to alleviate such stress, even though both are protein synthesis inhibitors (24). This difference is presumably because their modes of action are different, impacting different steps of protein translation, as no biased effects on the translation of individual mRNAs were observed (24). Furthermore, the possible consequences of off-target effects may be another contributing factor. For example, ANM has been well characterized to strongly activate the mitogen-activated protein (MAP) kinases JNK/SAPK (c-Jun NH2-terminal kinase/stress-activated protein kinase) in mammalian cells, resulting in rapid induction of immediate-early gene expression (46). The underlying mechanisms behind the different inhibition efficiencies by different inhibitors remain to be further explored.

The ribosome is the target of these three inhibitors, even though their inhibition mechanisms are different. The ribosome biogenesis under the inhibitor treatment may be related to the resistance of the inhibition, which further affects the overall inhibition efficiency. GO enrichment analysis revealed that the cytosolic large ribosomal subunits were significantly less sensitive to CHX than both ANM and Puro (Fig. 6A and Dataset S9). For Puro, it had poorer inhibitions on the cytosolic large ribosomal subunits than ANM. The results demonstrated that CHX exhibited significantly lower efficiencies on ribosomal proteins in three types of cells. In MCF7 cells, CHX had similar efficiencies with Puro and was significantly less efficient than ANM (Fig. 6B). Generally, ribosomal proteins are more sensitive to the inhibition than the whole proteome (Figs. 2A and S2). We then separately analyzed the inhibition efficiencies of the cytosolic/mitochondrial and large/small ribosomal subunits (Dataset S10). The mitochondrial ribosomal subunits were generally less inhibited than the cytosolic ones. The cytosolic ribosomal large subunits displayed significantly higher inhibition resistance to CHX than Puro and ANM, and to a lesser extent, the cytosolic ribosomal small subunits were more resistant to CHX (Figs. 6B and S6B).

Together, these results indicated that CHX was relatively less effective in inhibiting cytosolic ribosomal proteins than Puro and ANM, typically the cytosolic large ribosome subunits. One possible reason is that the CHX treatment can rapidly accumulate mRNAs related to ribosome biogenesis (25, 47), which may enhance cytosolic ribosome translation, thus diminishing the inhibition efficiencies of ribosomal proteins. Notably, in those studies, global translation inhibition was reported to be the reason for ribosome biogenesis-related mRNA accumulation. Considering the inhibition efficiency differences of ribosomal proteins by CHX and the other two inhibitors, the current results raise a question regarding if the reported ribosome biogenesis regulation was due to global protein synthesis inhibition or the CHX-specific effects, which requires further investigation.

## Discussion

The inhibition of protein synthesis is frequently utilized to unravel protein functions and cellular activities (7, 32). Although protein synthesis inhibitors have been widely investigated and employed in many studies (2–4), it is extraordinarily challenging to systematically study and evaluate their inhibition efficiencies, especially for individual proteins by each inhibitor. In this work, we comprehensively quantified and evaluated the inhibition efficiencies of individual proteins by three commonly used inhibitors through global quantification of newly synthesized proteins. Because newly synthesized proteins are the immediate and direct products of protein synthesis by the ribosome, comprehensive and quantitative analysis of them provides us a unique opportunity to study protein synthesis inhibition and evaluate different inhibitors. Besides protein synthesis inhibition, previous studies demonstrated that different inhibitors may have varying biological outcomes. For example, it was reported that ANM was more efficient in facilitating embryo development to blastocyst than CHX (48). Another example is that ANM and CHX were found to inhibit tunicamycin-induced unfolded protein response in HeLa cells, but Puro could not (49). The current work may help us understand the underlying mechanisms of these effects.

By integrating metabolic labeling and bioorthogonal chemistry, newly synthesized proteins were selectively enriched, allowing for their comprehensive analysis by MS while avoiding the masking effect from many highly abundant existing proteins (18–22). Moreover, a boosting approach was utilized to further increase the detection of newly synthesized proteins (50–52), especially low-abundance ones. In this work, we quantified >5,000 newly synthesized proteins and their synthesis inhibition efficiencies. The synthesis inhibition of proteins in four types of cells by each of three inhibitors was performed. The results clearly demonstrate that the inhibition efficiencies of individual proteins in the same type of cells by the same inhibitor are dramatically different, and the inhibition efficiencies of the same proteins by one inhibitor can also be very different in various cell types.

The inhibition efficiencies of proteins in different cellular compartments and protein complexes were systematically investigated in cells with the treatment of each inhibitor. For instance, nucleolar and ribosomal proteins are significantly more inhibited in every treatment, but proteins in the Golgi and endosome are less inhibited. The rRNA in the ribosome is generated in the nucleolus (34), and the ribosome biogenesis is critical in protein synthesis regulation (25, 47). The current results revealed that proteins involved in the ribosome biogenesis had higher inhibition efficiencies than the median value of all quantified proteins by each inhibitor. Furthermore, for the inhibition of ribosome biogenesis, CHX displayed relatively lower efficiencies than Puro and ANM in all tested cell types.

In conclusion, we systematically quantified the inhibition efficiencies of individual proteins in four types of human cells by three inhibitors, which contain a wealth of valuable information about protein synthesis inhibition. The correlations between the inhibition efficiencies of individual proteins and their various physicochemical and biological properties were further studied. The functions and multiple properties of proteins intrinsically sensitive or resistant to the inhibition were compared. Among resistant proteins, they are more hydrophobic and have less disordered regions. Further analysis revealed that the protein abundances and the translation efficiencies determined by Ribo-Seq are positively correlated with the inhibition efficiencies of corresponding proteins (37, 38). Intriguingly, proteins related to protein folding, especially chaperons, are highly enriched among those resistant to the inhibition. Chaperons are essential in assisting the folding of newly synthesized proteins and the refolding of existing misfolded proteins. Chaperone proteins are less sensitive to the inhibition, which may be due to their necessity in maintaining cell viability. Protein structures are vulnerable and carefully monitored and regulated in cells. When protein synthesis is inhibited, it is vital to ensure the structures and activities of existing proteins, which primarily rely on chaperones. These data indicate that the resistance of proteins to the inhibition is related to their functions. Global quantification of newly synthesized proteins allows for the first systematic evaluation of the synthesis inhibition efficiencies of individual proteins by different inhibitors. Unprecedented and valuable information about protein synthesis inhibition is obtained, which advances our understanding of protein synthesis regulation and provides useful resources to the biological and biomedical research communities. Both the overall inhibition efficiencies of these inhibitors and the inhibition efficiencies for individual proteins by each inhibitor will be beneficial for scientists to choose a suitable inhibitor for their future research.

## Materials and methods

A summary of key methods is given below (see Supplementary Methods for details). Detailed methods for all experiments and analyses are included in the Supplementary material.

### Cell culture and lysis for proteomics analysis

Cells were cultured in normal media until they reached ∼70% confluency. Then cells were grown in the methionine- and lysine-depleted media for 30 min to deplete free methionine and lysine before being cultured in the AHA, heavy lysine containing media for 1.0 h. The concentrations of AHA and heavy lysine are the same as those of methionine and lysine in normal media. During the depletion of free lysine and methionine and cell labeling, cells were treated with CHX, Puro, ANM, or DMSO with a final concentration of 6.9 µm, 10.8 µm, 25.1 µm, and 0.1% (*v*/*v*), respectively. Labeled cells were then harvested and lysed. Two biological replicated experiments were performed for each treatment.

### Proteomics analysis by MS

The cell lysates were clarified by centrifugation at 24,000 *g* at 4°C for 10 min, and the supernatant was kept. The clarified lysates were incubated with DBCO-conjugated magnetic beads at 4°C overnight. Proteins were then reduced and alkylated. Nonspecifically bound proteins were removed by stringent wash, and enriched newly synthesized proteins were digested by Lys-C overnight at 37°C with shaking. Digested peptides were desalted and lyophilized before TMT labeling and pooling. Pooled samples were fractionated using high-pH reversed-phase high-performance LC (HPLC) into 25 fractions in 10 mm ammonium formate (pH = 10). The collected fractions were further purified by the StageTip method. Quantitative LC–MS/MS analysis was performed with reversed-phase HPLC coupled with an Orbitrap Elite mass spectrometer (Thermo Fisher Scientific).

## Supplementary Material

pgad168_Supplementary_Data

## Data Availability

The raw files generated by MS were deposited in the ProteomeXchange Consortium (Project accession: PXD035992).
