# Supplementary material for: Global quantification of newly synthesized proteins reveals cell type- and inhibitor-specific effects on protein synthesis inhibition
Source: PNAS Nexus. 2023 May 19;2(6):pgad168. doi: 10.1093/pnasnexus/pgad168 (PMC10235912; doi:10.1093/pnasnexus/pgad168)
Supplement: pgad168_Supplementary_Data [file pgad168_supplementary_data.zip › PNASNEXUS-PNASNEXUS-2023-00216-T-s01.pdf]

Supplementary Material for

Global quantification of newly synthesized proteins reveals cell type- and inhibitor-specific effects on protein synthesis inhibition

Kejun Yin[1,4], Ming Tong[1,2,4], Suttipong Suttapitugsakul[1,3], Senhan Xu[1], and Ronghu Wu[1]*

[1] School of Chemistry and Biochemistry and the Petit Institute for Bioengineering and Bioscience, Georgia Institute of Technology, Atlanta, Georgia 30332, USA

[2] Present address: Novo Nordisk R&D Centre, Beijing, China

[3] Present address: Department of Surgery, Beth Israel Deaconess Medical Center, Harvard Medical School, Boston, MA, USA.

[4] These authors contributed equally to this work

* Correspondence: ronghu.wu@chemistry.gatech.edu (R.W.)

This PDF file includes:

Figures S1 to S6

Supplementary text

References

Other supplementary materials for this manuscript include the following:

Datasets S1 to S10

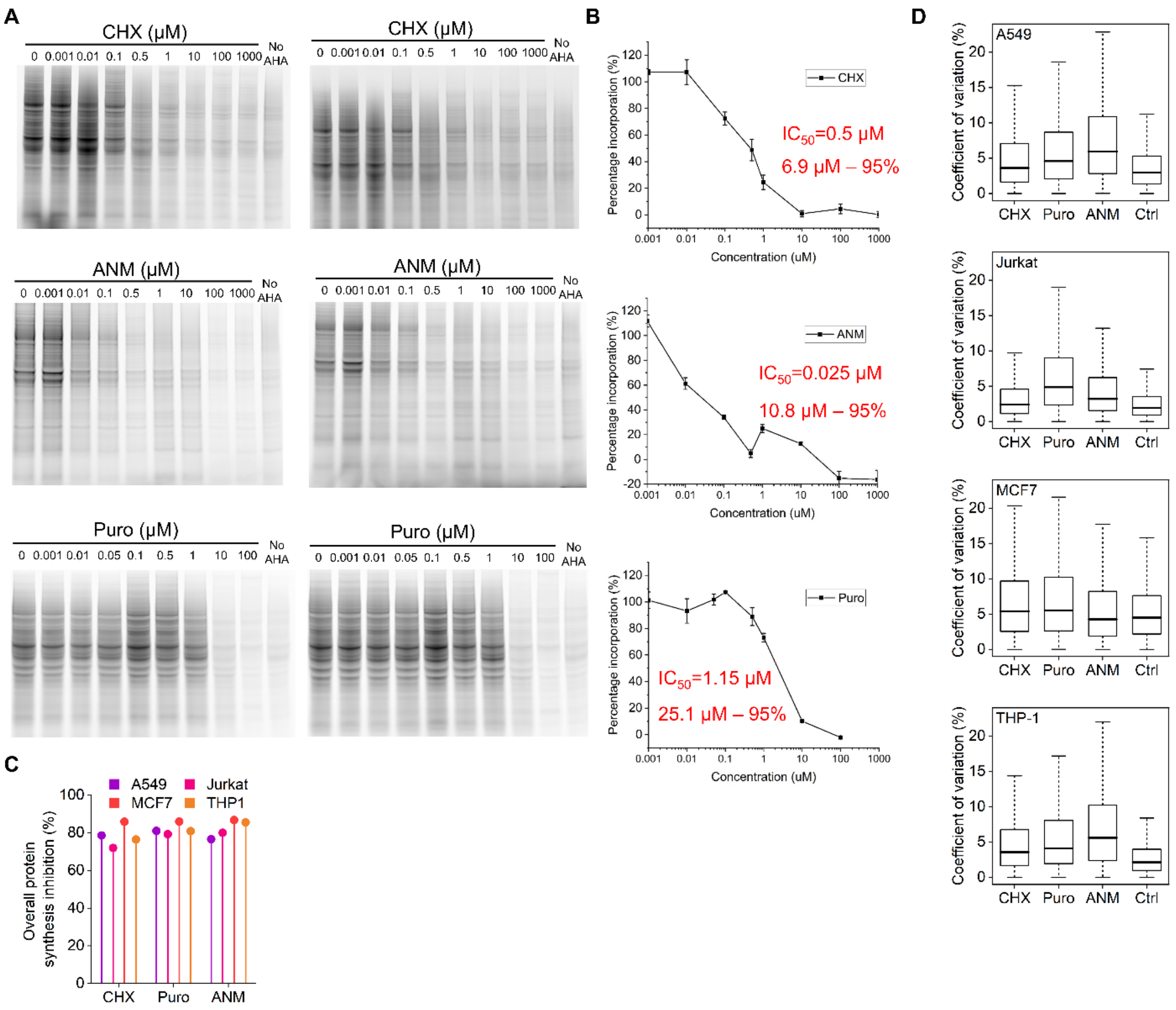

Fig. S1. Measurement of protein synthesis inhibition efficiencies. (**A**) Fluorescence results of newly synthesized proteins labeled with azidohomoalanine (AHA) under treatment with different concentrations of cycloheximide (CHX), puromycin (Puro), and anisomycin (ANM), respectively in MCF7 cells. The duplicate experiments were performed for each inhibitor. The total protein load was monitored through *Coomassie* blue staining. (**B**) Dose-dependent curves of the three tested inhibitors. The IC50 value and the concentration to reach 95% overall inhibition efficiency of total proteins were marked near the curve for each inhibitor. (**C**) The overall protein synthesis inhibition efficiencies for each inhibitor in all cell types measured in the MS-based proteomics experiments. The overall protein inhibition efficiencies are calculated based on the total intensity of the reporter ions for each treatment. (**D**) The coefficient of variation (CV) of the replicate results for each experiment with one inhibitor in one type of cells. A median CV of around 5% was observed for all experiments, demonstrating low variance within the replicates. Data are presented as box plots (center line: median; box limits: the first and third quartiles; whiskers: 1.5 interquartile range). **Related to Fig. 1.**

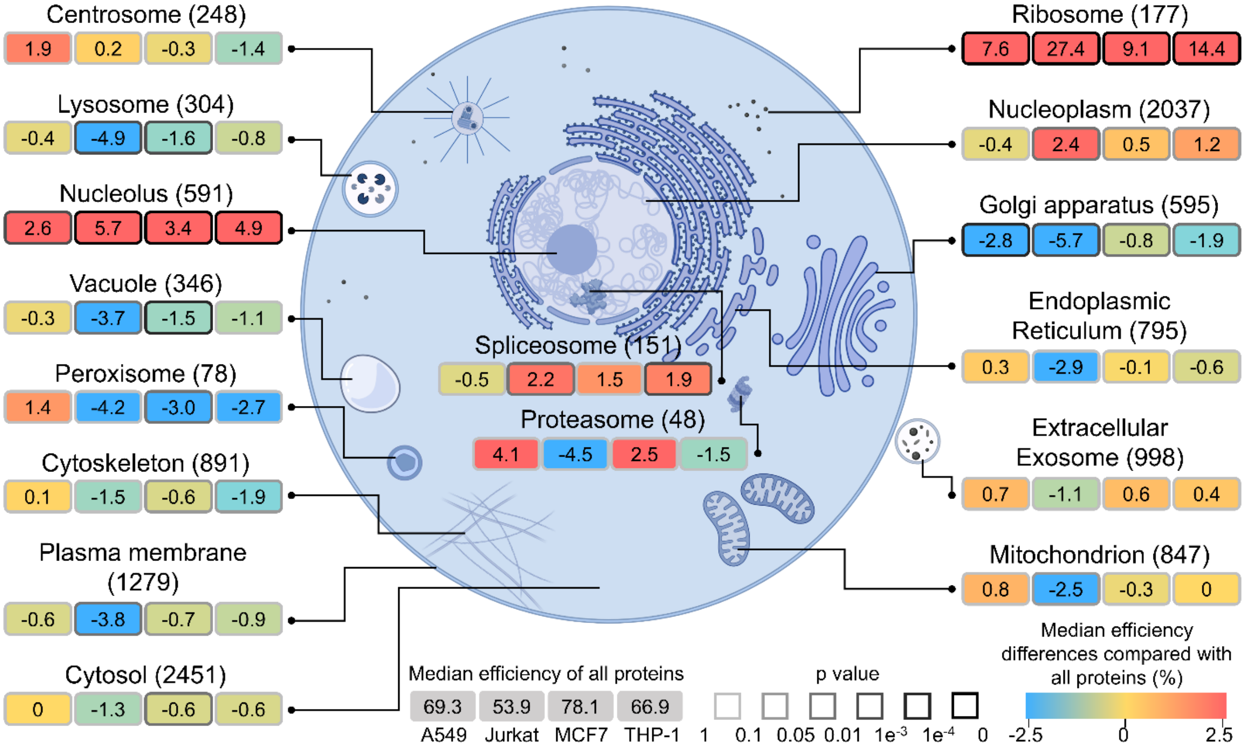

Fig. S2. The synthesis inhibition efficiencies of proteins in different cellular components and protein complexes among four cell types by CHX. The number of proteins quantified in each category is presented in the parentheses. The number in the box represents the median inhibition efficiency difference compared with the median value of all quantified proteins in each cell type. The color of the box also represents the difference of the inhibition efficiencies in each cell type. The borders of box indicate the significance of the difference (determined by the Kolmogorov-Smirnov test). **Related to Fig. 2.**

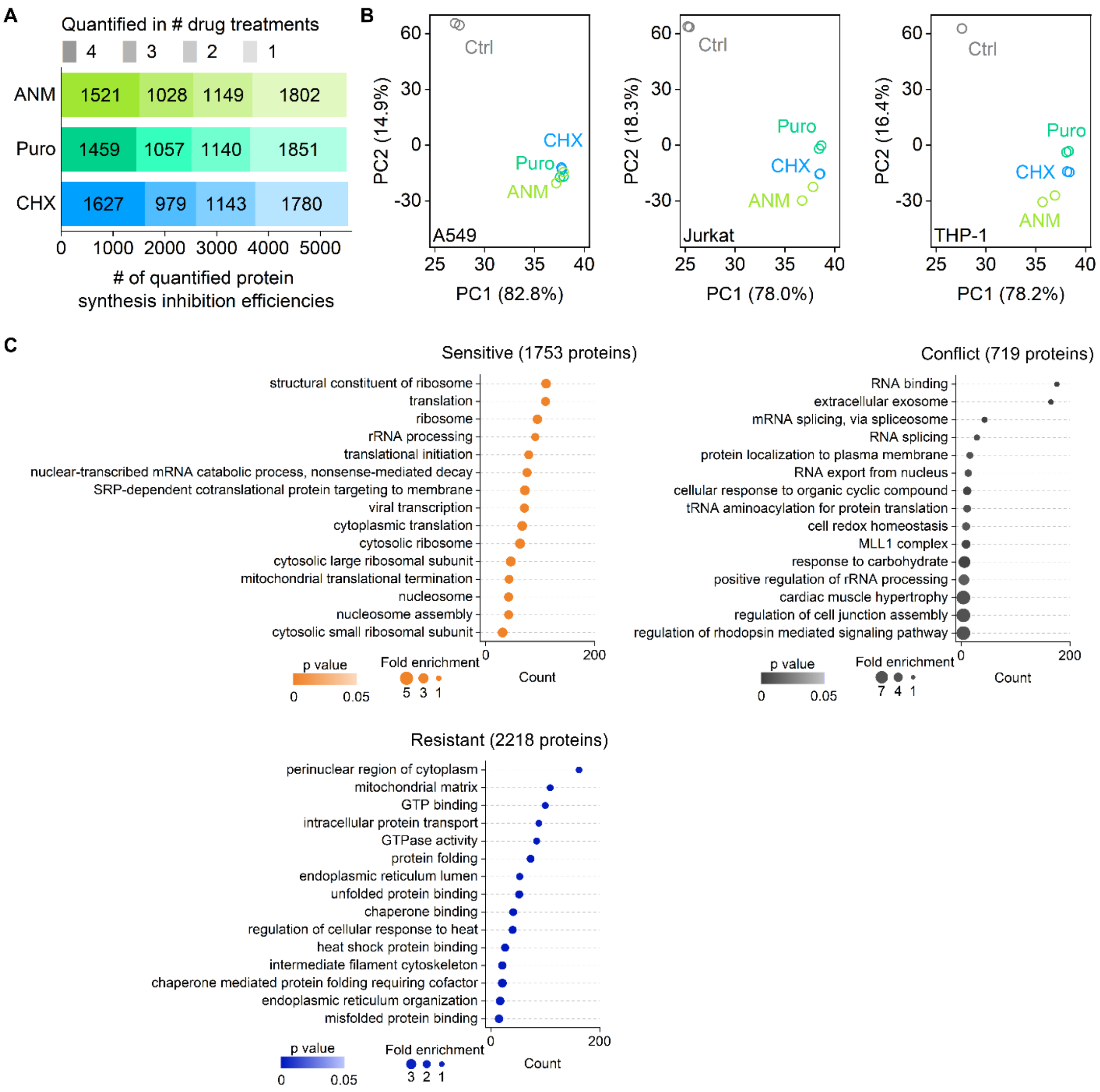

Fig. S3. Characterization of synthesis inhibition efficiencies in multiple types of cells. (A) The number of quantified protein synthesis inhibition efficiencies in the inhibitor treatment experiments. (B) Principle component analysis on the replicate samples treated with each inhibitor and the control samples. (C) Gene Ontology (GO) analysis on proteins that were annotated as sensitive only (orange), conflict (gray), and resistant only (blue). Only top 15 enriched terms are included. **Related to Fig. 3.**

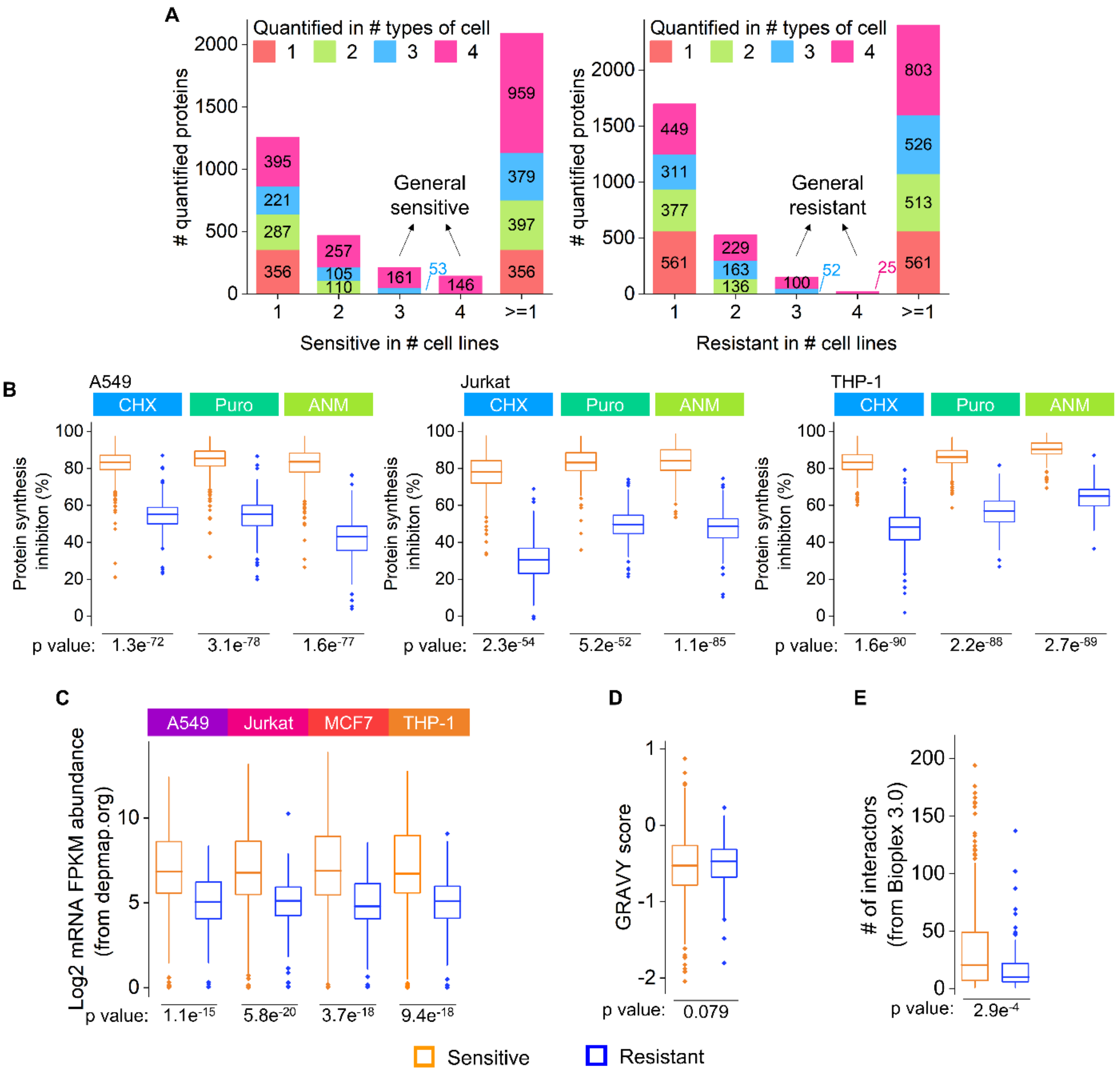

Fig. S4. Analysis on proteins generally sensitive/resistant to the synthesis inhibition. (A) Sensitive (left) or resistant (right) proteins shared by different cell types. For example, the second bar in the left chart means that 472 proteins were sensitive in at least two cell types. Among 472 proteins, 257 proteins were quantified in all four cell types, 105 proteins were quantified in three cell types, and 110 were quantified in two cell types. (B) Synthesis inhibition efficiency distributions of proteins sensitive or resistant to the inhibition in A549, Jurkat, and THP-1 cells. (C) Distributions of the mRNA abundances for corresponding proteins sensitive and resistant to the inhibition. The mRNA abundances in fragments per kilobase of transcript per million mapped reads (FPKM) are from the DepMap Portal. (D) Comparisons of the hydrophobicity between proteins sensitive and resistant to the synthesis inhibition. (E) Comparisons of the number of protein-protein interactors between proteins

sensitive and resistant to the synthesis inhibition (from Bioplex 3.0). (Centerline, median; box limits, the first and third quartiles; whiskers, 1.5 interquartile range. Statistical significance was determined by the K-S test.) **Related to Fig. 4.**

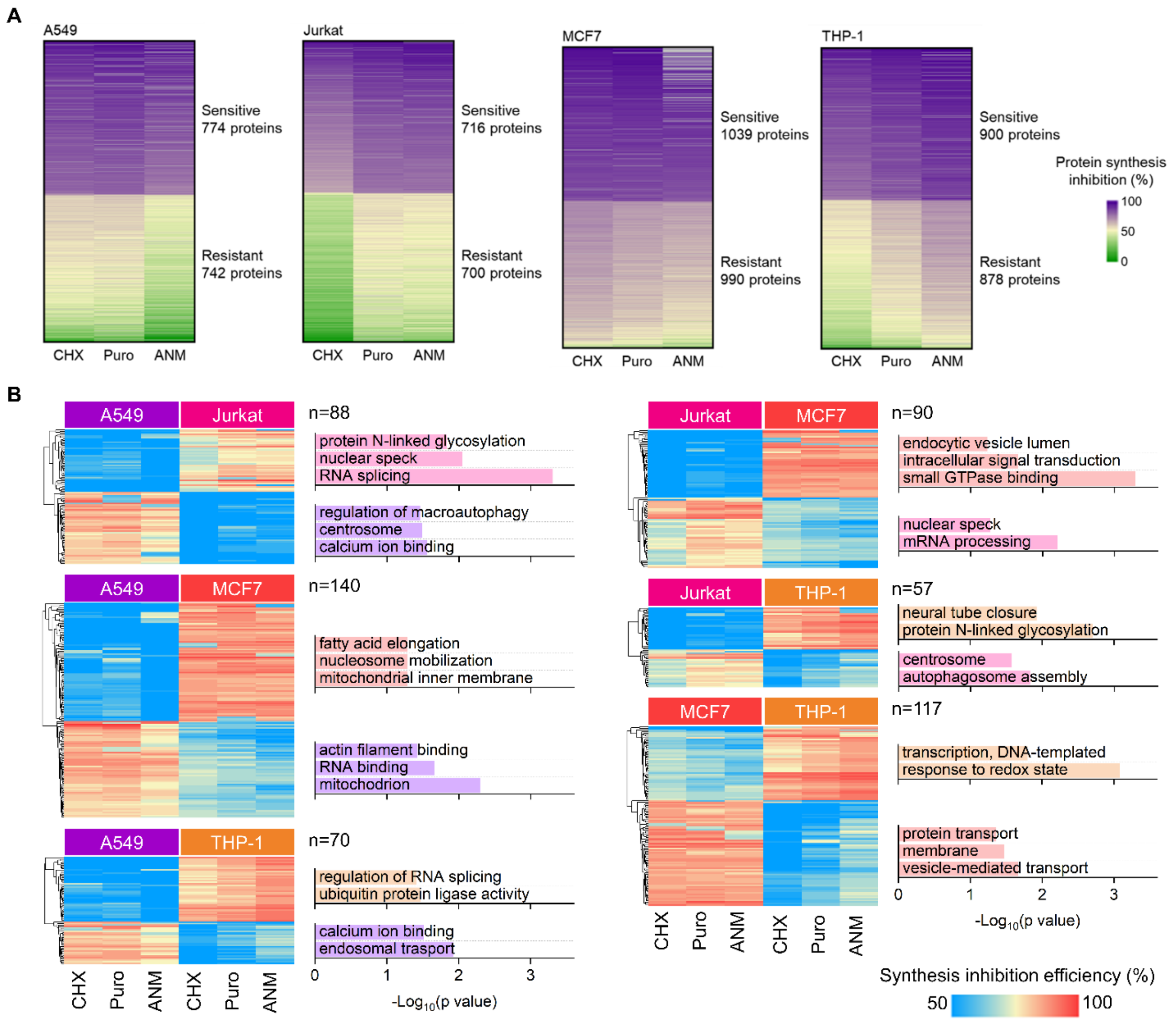

Fig. S5. Cell type-specific differences in the inhibition sensitivity of proteins. (A) Heatmaps of proteins being annotated as sensitive or resistant in each cell type. (B) A representative view of proteins showing different sensitivity annotations in two different cell types. Each group of proteins was submitted to GO enrichment analysis with all quantified proteins as the background. **Related to Fig. 5.**

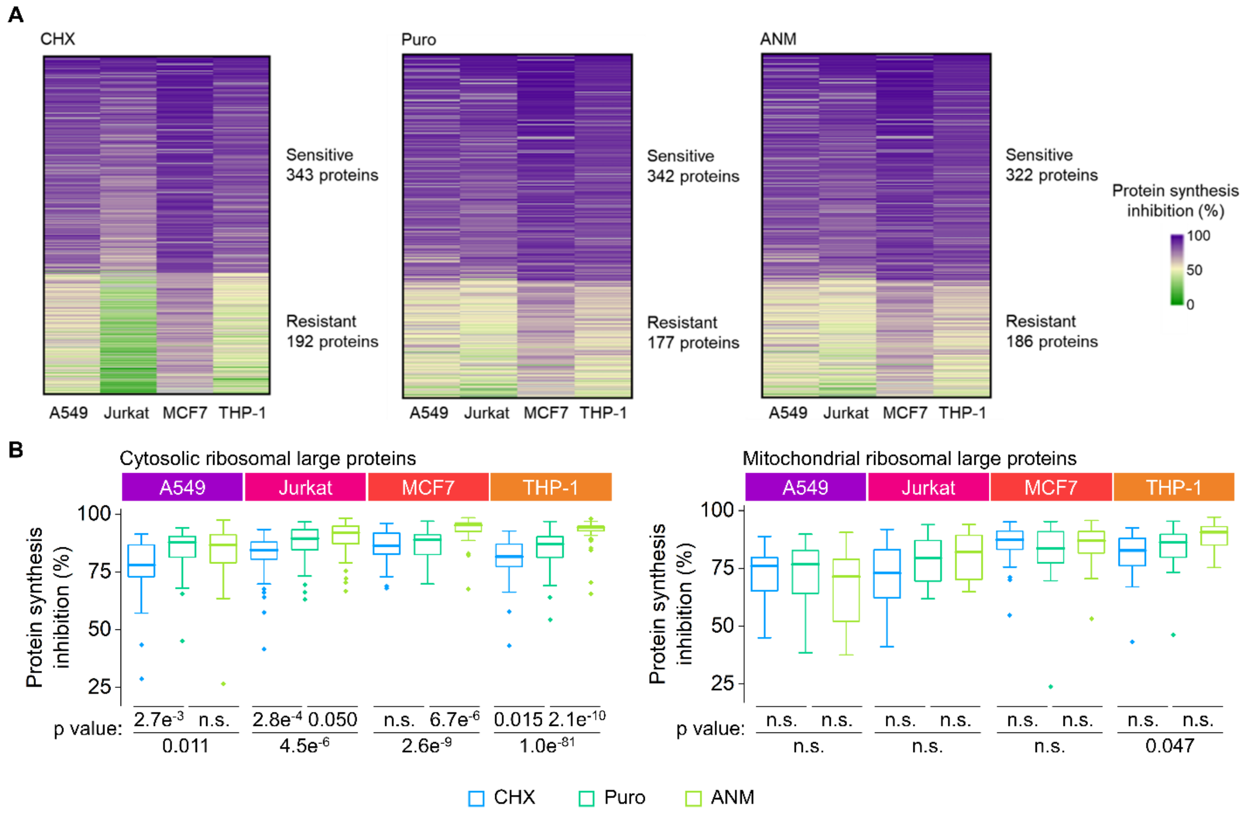

Fig. S6. Inhibitor-specific differences in the inhibition sensitivity of proteins. (A) Heatmaps of proteins being annotated as sensitive or resistant to each inhibitor. (B) Comparison of protein synthesis inhibition efficiencies of different ribosomal subunits in different experiments. (Centerline, median; box limits, the first and third quartiles; whiskers, 1.5 interquartile range. Statistical significance was determined by the K-S test (two-sided). "n.s." means p value > 0.1). **Related to Fig. 6.**

Supplementary Methods

Metabolic labeling and cell treatment with protein synthesis inhibitors

A549 and MCF-7 cells (American Type Culture Collection (ATCC)) were cultured in Dulbecco's modified eagle's medium (DMEM) (Sigma-Aldrich) with 10% fetal bovine serum (FBS) (Corning). Jurkat and THP-1 cells (American Type Culture Collection (ATCC)) were cultured in Roswell Park Memorial Institute (RPMI) 1640 medium (Sigma-Aldrich) with 10% FBS (Corning). All cells were cultured in an incubator with 5% CO_2 at 37 °C.

For A549 and MCF7 cells, they were grown in T-175 flasks (SPL life science) until ~70% of confluency was reached. Then all cells were detached, washed with phosphate buffered saline (PBS) (Sigma-Aldrich), and equally split into two 6-well plates (Corning) for the gel-based experiments, and into eight T-75 flasks for the MS-based experiments. Cells were then cultivated in the normal DMEM medium. For Jurkat and THP-1 cells, they were cultivated in the RPMI medium until their density reached 10^6 cells/mL. Then all cells were washed with PBS and equally split into eight T-75 flasks. After 48 h, cells were starved by replacing the medium with Lys and Met depleted DMEM (Athena) (for MCF7 and A549 cells) or RPMI (Athena) (for Jurkat and THP-1 cells) and 10% dialyzed FBS (DiFBS, R&D Systems) for 30 min before metabolic labeling. For the gel-based experiments, cycloheximide (CHX), puromycin (Puro), anisomycin (ANM), and dimethyl sulfoxide (DMSO) (Sigma-Aldrich) was added to each well, respectively, to the desired final concentration at the same time. For the MS-based experiments, CHX, Puro, ANM, and DMSO were added to the corresponding flasks to a final concentration of 6.9 µM, 10.8 µM, 25.1 µM, and 0.1%, respectively.

Cells were metabolically labeled in the depleted DMEM containing heavy lysine (13C6 and 15N2, +8 Da, Lys8) (Cambridge Isotope Laboratories), azidohomoalanine (AHA) (Click Chemistry Tools), and 10% dialyzed fetal bovine serum (Fisher Scientific) for 1 hour with the same concentration of each inhibitor and DMSO, respectively. The concentration of heavy lysine and AHA were the same as the concentration of lysine and methionine in normal DMEM (0.8 mM and 0.2 mM, respectively) and RPMI (0.2 mM and 0.1 mM, respectively). For the booster channel, cells were metabolically

labeled with heavy lysine and AHA for 48 h. Then the cells were collected, and the cell numbers were counted using a hemocytometer.

Cell lysis

Cells were pelleted using 300g centrifugation for 5 min. The cell pellets were washed with ice-cold PBS twice, and lysed with end-over-end rotation at 4°C for 1 hour in the lysis buffer containing 50 mM 4-(2-hydroxyethyl)piperazine-1-ethanesulfonic acid (HEPES) pH=7.4, 150 mM NaCl, 1 mM $MgCl_2$, 0.5% sodium deoxycholate (SDC), benzonase (10 Unit/mL) and the cOmplete protease inhibitor cocktail (Roche). The lysates were clarified at 24,000 g at 4°C for 10 min before the following steps.

Measurement of the dose-dependent overall synthesis inhibition in MCF7 cells

The protein concentrations in the lysates from the treated MCF7 cells were determined using the BCA assay. Equal amounts of proteins were then transferred into new PCR tubes according to the results from the BCA assay. Newly synthesized proteins were labeled by adding 1 µL DBCO-PEG4-Fluor 545 (Sigma-Aldrich) into each PCR tube and shaking the mixture for 4 hours at room temperature. The reaction was then quenched by adding 2 µL of 200 mM AHA solution. Proteins were separated using sodium dodecyl sulfate–polyacrylamide gel electrophoresis (SDS-PAGE), and then the fluorescence signals were detected.

Synthesis of DBCO-conjugated magnetic beads

The beads were synthesized by mixing the amine derivatized MagnaBind beads (Fisher Scientific) with DBCO-sulfo-N-hydroxysuccinimide (NHS) (Click Chemistry Tools) in DMSO overnight. The beads were washed with the lysis buffer prior to being used for the enrichment.

Newly synthesized protein enrichment and protein digestion

The supernatants of the lysates were transferred to new tubes and incubated with dibenzocyclooctyne (DBCO) conjugated magnetic beads at 4 ˚C overnight. Newly synthesized proteins bearing the azido

group were enriched through the click reaction between the azido and DBCO groups. Enriched proteins were then reduced with 5 mM dithiothreitol (DTT) at 56 °C for 25 min and subsequently alkylated with 15 mM iodoacetamide (IAA) at room temperature for 30 min in the dark. The beads were washed with 2 mL lysis buffer with 2.5% sodium dodecyl sulfate (SDS) and 2.5% SDC four times at 95°C, 2 mL of 8 M urea in 100 mM HEPES pH=8.1 three times, 2 mL 50% isopropanol twice, and 2 mL of 50% acetonitrile (ACN) twice. The magnetic beads were resuspended in the digestion buffer containing 50 mM HEPES pH=8.1, 1.6 M urea, and newly synthesized proteins were digested with 100:1 (w/w) lysyl endopeptidase (Lys-C) (Wako) overnight at 37 ˚C with shaking. The digestion was quenched by adding trifluoroacetic acid (TFA) to a final concentration of 0.4%. Digests were centrifuged to remove the precipitates and were desalted using 50 mg SepPak tC18 cartridges (Waters).

TMT labeling and peptide fractionation by HPLC

Purified peptides were lyophilized and resuspended in 35 μL of 100 mM HEPES pH = 8.5, and 10 μL ACN. For each cell type, the samples were labeled with the TMT10plex reagents (Thermofisher). After the one-hour reaction, the excessive amount of the TMT reagents was quenched by adding 10 μL of 5% hydroxylamine. Labeled peptides in the same group were pooled, purified, and fractionated by high-pH reversed-phase high performance liquid chromatography (HPLC) into 25 fractions with a 40-min gradient of 5-50% ACN in 10 mM ammonium formate (pH=10). The collected fractions were further purified by the StageTip method.

LC-MS/MS analysis

Dried peptides were resuspended in 6 µL loading solution containing 5% ACN and 4% formic acid (FA), and 3 µl was loaded onto a microcapillary column packed with C18 beads (Magic C18AQ, 5 µm, 200 Å, 75 µm x 16 cm) by a Dionex WPS-3000TPLRS autosampler (UltiMate 3000 Thermostatted Pulled Loop Rapid Separation Wellplate Sampler). Peptides were separated by reversed-phase HPLC using an UltiMate 3000 binary pump with a 150-minute gradient designed for the TMT-labeled peptides.

The full MS and MS2 were detected in a hybrid dual-cell quadrupole linear ion trap – Orbitrap mass spectrometer (LTQ Orbitrap Elite, Thermo Scientific, with Xcalibur 3.0.63 software) using a data-dependent Top15 method. Each cycle included one full MS scan (resolution: 60,000) in the Orbitrap at the automatic gain control (AGC) target of $1*10^6$, followed by up to 15 MS/MS for the most intense ions. Selected ions were excluded from further analysis for 90 s. Ions with a single or unassigned charge were not sequenced. MS2 scans were activated by higher-energy collisional dissociation (HCD) at 40% normalized collision energy with an isolation width of 1.2 m/z and detected in the Orbitrap cell with a resolution of 30,000.

Database searching, data filtering, and protein quantification

Raw data files recorded from the mass spectrometer were converted into the mzXML format. Mass spectra were searched using the SEQUEST algorithm (version 28) (1) against a human proteome (Homo sapiens) database encompassing sequences of all proteins downloaded from UniProt (https://www.uniprot.org/taxonomy/9606). Each protein sequence was listed in both forward and reversed orientations to estimate the false discovery rate (FDR) of peptide and protein identifications. The following parameters were used for the search: 10 ppm precursor mass tolerance; 0.025 Da product ion mass tolerance; fully digested with Lys-C; up to two missed cleavages; variable modifications: oxidation of methionine (+15.9949) and heavy lysine (+8.0142); fixed modifications: carbamidomethylation of cysteine (+57.0214) and the TMT labeling of lysine and the N-termini (+229.1630).

The target-decoy method (2) was employed to evaluate and control the FDRs of peptide and protein identifications. Linear discriminant analysis (LDA) was used to distinguish correct and incorrect peptide identifications using numerous parameters such as XCorr, ΔCn, and precursor mass error. After scoring, peptides with fewer than seven amino acids in length were discarded, and peptides were filtered to a less than 1% FDR based on the number of decoy sequences in the final data set. Afterward, the FDR is further controlled to <1% at the protein level.

The TMT reporter ion intensities in the MS2 spectra were used to quantify peptides. The isotopic information provided by Thermo was utilized to correct the ion intensities. The summed intensity for each protein was calculated based on all unique peptides from this protein in each sample. The average intensity of each duplicate was calculated, and the protein ratio under the treatment of each inhibitor was calculated as the ratio of the average intensity with the treatment to the average intensity in the control samples (DMSO). The inhibition efficiency of each protein was calculated by subtracting the protein ratio from one. The overall inhibition efficiency for each inhibitor was calculated by subtracting the ratio of the total ion intensity with the treatment to the total ion intensity in the control sample from one. Inhibition efficiencies between two replicated experiments showing reliable reproducibility (i.e., coefficient of variance (CVs) < 20%) were remained. It should be noted that some proteins were upregulated during the inhibition, causing them to have a negative inhibition efficiency. We manually assigned their efficiencies as zero after filtering of CVs as mentioned above. This did not affect the following data analysis because less than 20 out of thousands of proteins were found to have a negative efficiency in each treatment.

Bioinformatic analysis

Protein functional annotation information was obtained from UniProt (3) (https://www.uniprot.org/) and analyzed using the Database for Annotation, Visualization and Integrated Discovery (4) (DAVID, https://david.ncifcrf.gov/) using all identified newly synthesized proteins as the background unless mentioned otherwise. The illustrations of subcellular compartments analysis were generated by BioRender (https://app.biorender.com/biorender-templates). Protein abundance data extracted from PAXdb (https://pax-db.org/) (5). Baseline mRNA levels of each cell type were obtained from Depmap (https://depmap.org/portal/). Pre-computed protein isoelectric points were obtained from Proteome-pI: Proteome Isoelectric Point Database (6) (http://isoelectricpointdb.org/index.html). Protein disordered regions were predicted by Regression-based Accurate Prediction of protein Intrinsic Disorder (RAPID) webserver (7) (http://biomine.cs.vcu.edu/servers/RAPID/). Protein-protein interaction data extracted

from Bioplex 3.0 (https://bioplex.hms.harvard.edu/index.php) (8). Statistical analysis was performed using Excel and OriginPro 2022. The statistical details of the experiments can be found in the Results section and in the captions of corresponding Figures.

References

1. J. K. Eng, A. L. McCormack, J. R. Yates, An approach to correlate tandem mass spectral data of peptides with amino acid sequences in a protein database. *J. Am. Soc. Mass Spectrom.* **5**, 976-989 (1994).
2. J. E. Elias, S. P. Gygi, Target-decoy search strategy for increased confidence in large-scale protein identifications by mass spectrometry. *Nat. Methods* **4**, 207-214 (2007).
3. C. UniProt, UniProt: a worldwide hub of protein knowledge. *Nucleic Acids Res.* **47**, D506-D515 (2019).
4. W. Huang da, B. T. Sherman, R. A. Lempicki, Systematic and integrative analysis of large gene lists using DAVID bioinformatics resources. *Nat Protoc* **4**, 44-57 (2009).
5. M. Wang, C. J. Herrmann, M. Simonovic, D. Szklarczyk, C. von Mering, Version 4.0 of PaxDb: Protein abundance data, integrated across model organisms, tissues, and cell-lines. *Proteomics* **15**, 3163-3168 (2015).
6. L. P. Kozlowski, Proteome-pI 2.0: proteome isoelectric point database update. *Nucleic Acids Res.* **50**, D1535-D1540 (2022).
7. J. Yan, M. J. Mizianty, P. L. Filipow, V. N. Uversky, L. Kurgan, RAPID: fast and accurate sequence-based prediction of intrinsic disorder content on proteomic scale. *Biochim. Biophys. Acta.* **1834**, 1671-1680 (2013).
8. E. L. Huttlin *et al.*, Dual proteome-scale networks reveal cell-specific remodeling of the human interactome. *Cell* **184**, 3022-3040 (2021).
9. B. Gyorffy, Survival analysis across the entire transcriptome identifies biomarkers with the highest prognostic power in breast cancer. *Comput. Struct. Biotechnol. J.* **19**, 4101-4109 (2021).
10. B. Gyorffy, P. Surowiak, J. Budczies, A. Lanczky, Online survival analysis software to assess the prognostic value of biomarkers using transcriptomic data in non-small-cell lung cancer. *PLOS One* **8**, e82241 (2013).
11. A. Bartha, B. Gyorffy, TNMplot.com: a web tool for the comparison of gene expression in normal, tumor and metastatic tissues. *Int. J. Mol. Sci.* **22**, 2622-2633 (2021).
